# Successful Applicant and Program Director Perspectives on the Virtual Residency Selection Process for Canadian Surgical Subspecialties

**DOI:** 10.1177/22925503221108468

**Published:** 2022-07-05

**Authors:** Jad Abi-Rafeh, Victoria Sebag, Hassan ElHawary, Dino Zammit, Mirko S. Gilardino

**Affiliations:** 1 Division of Plastic and Reconstructive Surgery, 54473 McGill University Health Center, Montreal, Quebec, Canada; 2 12367 Faculty of Medicine, McGill University, Montreal, Quebec, Canada

**Keywords:** surgical training, residency, CaRMS, virtual, interviews, clinical electives

## Abstract

**Introduction:** The COVID-19 pandemic imparted an important shift in strategies postgraduate surgical programs use to recruit, interact with, and select medical students applying through the Canadian Resident Matching Service (CaRMS). With this unprecedented shift toward virtual applicant selection, this study sought to explore and analyze perspectives of the first cohort of program directors (PDs) and applicants who participated in this process. **Methods:** A cross-sectional survey study was designed using Google Forms for both PDs and applicants participating in the 2021 CaRMS surgical subspecialty selection process. Questions pertained to format and content of virtual engagement methods, the interview itself, as well as advice for future applicants. **Results:** Thirty-five PDs and 40 successful applicants (n = 75) participated in the study. Cost reduction was the most commonly reported benefit of online interviewing by PDs (85%), followed by efficiency (71%), enhanced resource management (49%), and ability to conduct more interviews (23%). Strong letters of reference (80%) and interview performance (74%) remained the most significant factors in virtual applicant selection. Attendance to virtual recruitment events did not increase the likelihood of offering interviews (n = 24, 69% of PDs), although the ability to perform in-person electives held tremendous value. Most applicants (90%) reported on virtual information sessions as the best method for learning about programs; work culture and environment were topics most valued as discussion points (90%). Successful applicants provided an average confidence of 76% regarding their suitability with their matched programs. Seventy-three percent of applicants (n = 29) had either a preference for virtual interviews or were equivocal, while 51.4% of PDs (n = 18) preferred interviews to be conducted virtually for future cohorts. **Conclusion:** Trainees are entering residency with confidence following a virtual selection process, and PDs feel confident in their selections. Although no clear consensus exists regarding preference for virtual or in-person interviews, several advantages for virtual resident selection exist. The influence of an in-person elective was found difficult to replace, regardless of interview format. The importance of applicant engagement with programs prior to interviews is highlighted and discussed with recommendations provided for best practices.

## Résumé

**Introduction:** La pandémie de COVID-19 a induit un important changement de stratégies de l’utilisation des programmes chirurgicaux de 3^e^ cycle pour recruter, interagir avec les étudiants en médecine et sélectionner les étudiants déposant une candidature par le biais du CaRMS, le service d’affectation des résidents canadiens. Avec ce mouvement sans précédent vers une sélection virtuelle des candidats, l’étude a cherché à explorer et analyser les points de vue de la première cohorte de directeurs de programmes (DP) et de candidats ayant participé à ce processus. **Méthodes:** Une enquête transversale a été conçue à l’aide de Google Forms pour, à la fois, les DP et les candidats participant au processus de sélection de sous-spécialité chirurgicale 2021 du CaRMS. Des questions portaient sur le format et le contenu des méthodes de contact virtuelles, l’entretien proprement dit ainsi que sur des conseils pour les candidats futurs. **Résultats:** Trente-cinq DP et quarante candidats acceptés (n = 75) ont participé à l’étude. La réduction des coûts a été l’avantage des entretiens en ligne le plus souvent cité par les DP (85 %) suivie de l’efficacité (71 %), d’une meilleure gestion des ressources (49 %) et de la capacité à réaliser plus d’entretiens (23 %). De solides lettres de recommandation (80 %) et la performance au cours de l’entretien (74 %) restaient les facteurs les plus significatifs pour la sélection virtuelle des candidats. La participation aux événements virtuels de recrutement n’augmentait pas la probabilité d’offre d’un entretien (n = 24, 69 % des DP), même si la possibilité de réaliser des entretiens facultatifs en personne avait une valeur considérable. La plupart des candidats (90 %) ont indiqué que les séances d’information virtuelles étaient la meilleure méthode pour en savoir plus sur les programmes; la culture et l’environnement de travail étaient les thèmes ayant le plus de valeur comme sujets de discussion (90 %). Les candidats ayant réussi ont indiqué à 76 % qu’ils s’estimaient confiants sur le fait de bien correspondre aux programmes auxquels ils étaient affectés. Soixante-treize pour cent des candidats (n = 29) avaient une préférence pour les entretiens virtuels ou étaient dans le doute, tandis que 51,4 % des DP (n = 18) préféraient que les entretiens des cohortes futures soient menés virtuellement. **Conclusion:** Les stagiaires commencent leur résidence avec confiance après un processus de sélection virtuel et les DP se sentent confiants quant à leurs choix. Même s’il n’existe pas un franc consensus concernant une préférence pour les entretiens virtuels ou en face à face, la sélection à distance des résidents présente plusieurs avantages. L’influence d’un entretien optionnel en personne s’est avérée difficile à remplacer, quelle que soit la forme donnée à l’entretien. L’importance de l’engagement du candidat dans des programmes antérieurs aux entretiens est soulignée et discutée avec des recommandations fournies pour de meilleures pratiques.

## Introduction

In 2020, the COVID-19 pandemic forced medical educators and trainees to find alternative methods of granting exposure to surgical residency programs in Canada.^1^ With limited electives and shadowing opportunities, programs began organizing virtual information sessions, social events, and the Canadian Resident Matching Service (CaRMS) interviewing process shifted from traditional in-person interviews to an online format.^2^ This shift brought about new challenges for both medical students and program directors alike.

Since medical trainees represent the future of medicine and surgery, it remains critical to evaluate and improve upon the recruitment methods used. Since the CaRMS process was newly made virtual as of the 2021 application cycle, there exist a paucity of data with regard to optimal factors affecting virtual engagement and Canadian surgical applicant selection. Although some of these factors have been studied in the United States, to the authors’ knowledge, this has not yet been studied in Canada.^3^

In the present study, the authors aim to analyze the perspectives of both applicants and program directors who had participated in the first iteration of the virtual CaRMS surgical residency selection process, with the overarching goal of providing guidelines and recommendations for its sustainable adoption in future years.

## Methods

This study was designed as a cross-sectional survey study sent to trainees and program directors (PDs) who participated in the 2021 CaRMS surgical program application cycle. All surgical subspecialties were included. PD contact information was available publicly through the Royal College of Physicians and Surgeons of Canada website, and recent medical school graduates were reached through their program directors, postgraduate medical education administrators, or medical school council representatives. Institutional Review Board ethics approval was obtained (review number A07-E38-21B 21-07-078) prior to study commencement. Participation was voluntary and anonymous; all participants signed an informed consent form at the questionnaire offset. Questionnaire details are provided in Supplemental materials (Supplemental Digital Content 1-3).

## Results

### Program Director Feedback

#### Demographics

Thirty-five out of 128 Canadian surgical program directors (27%) responded to the survey, the majority of which from Quebec (n = 13, 37%) and Ontario (n = 7, 20%). The majority of PDs were from orthopedic surgery programs (n = 6, 17%), followed by general surgery (n = 5, 14%), urology (n = 5, 14%), plastic surgery (n = 4, 11%), ophthalmology (n = 4, 11%), otolaryngology (n = 4, 11%), cardiac surgery (n = 4, 11%), neurosurgery (n = 2, 6%), and vascular surgery (n = 1, 3%); complete demographic data is presented in Table 1. A percentage comparison of program directors who responded to the survey, relative to the total number of surgical program directors per province, is presented as Supplemental Digital Content 4.

**Table 1.** Breakdown of Program Director Demographics and Interaction Methods.

		Number of PDs (n)	%
Province	Quebec	13	37
	Ontario	7	20
	Alberta	5	14
	Saskatchewan	4	11
	Nova Scotia	3	9
	British Columbia	2	6
	Manitoba	1	3
Surgical Program	Orthopedic Surgery	6	17
	General Surgery	5	14
	Urology	5	14
	Plastic Surgery	4	11
	Ophthalmology	4	11
	Otolaryngology/Head/Neck	4	11
	Cardiac Surgery	4	11
	Neurosurgery	2	6
	Vascular Surgery	1	3
Methods used to recruit and interact with potential applicants	Virtual information sessions	30	86
	Active program website	25	71
	Open communication between PDs and applicants via email	20	57
	Virtual social events with residents	18	51
	Active social media pages	15	43
	Phone calls with interested applicants	1	3
	Open communication with residents	1	3

#### Perceived advantages

Cost reduction was the most commonly reported benefit of online interviewing perceived by PDs (n = 30, 85%), followed by efficiency (n = 25, 71%), enhanced resource management and planning (n = 15, 49%), as well as ability to conduct a greater number of interviews than usual (n = 8, 23%). Three PDs added that virtual interviews allowed the inclusion of faculty that may have otherwise been out of the country or physically unavailable, while one other PD (3%) reported on the benefit for applicants as it pertains to less travel.

#### Perceived disadvantages

The most commonly reported disadvantages of the virtual interview process included the inability to show applicants the program and facilities, as well as the inability to socialize with applicants to gauge their interpersonal traits (n = 24 each, 69%). Six PDs (17%) reported that they found themselves interviewing more applicants than truly necessary, while 2 other PDs reported on the difficulty of virtual event planning, and difficult applicant–interviewer interactions in the virtual setting (3% each). Overall, 54.3% of PDs (n = 19) felt that the benefits of online interviewing outweighed its disadvantages.

#### Program director outlook

Overall, 31% of PDs felt that they were very capable of representing their programs using virtual methods. When queried about their confidence in the fit of incoming residents to their programs, 49% responded that they were “very” confident, and 41% responded that they were “somewhat” confident. When queried about whether they would prefer virtual or in-person interviews for future cohorts, results were equivocal; 18 PDs (51%) preferred a virtual format and 17 (49%) preferred in-person. Seven PDs further elaborated that regardless of the interview format, nothing could replace the value of an in-person elective as a major factor in gauging applicant suitability, and ultimately, resident selection (n = 7, 20%). It remained unclear whether the lack of clinical electives specifically, or the overall virtual process, had the most impact on PD-perceived satisfaction presented herein.

When queried about factors that made applicants from outside schools appealing given the absence of visiting electives, the majority of PDs reported that “strong letters” (n = 28, 80%) and “strong interviews” (n = 26, 74%) were the most significant factors. Extracurriculars outside of medicine (n = 15, 43%), leadership positions (n = 14, 40%), and research experience (n = 12, 34%) were also highly valued. The majority of PDs (n = 24, 69%) reported that attendance to virtual information sessions did not increase the likelihood of an applicant being invited to an interview. Two PDs (6%) further emphasized the importance of interacting well with the interview panel and treating the virtual interview as though it was in-person. Four PDs (11%) suggested that applicants reach out to their programs (through residents, coordinators, or directly to PDs themselves) in order to demonstrate interest and learn about their programs prior to interviews. With regard to external factors such as lighting, clothing choice, and background during the virtual information sessions, socials, or interviews themselves, the majority of PDs reported that they did not play a significant role in applicant selection. Specifically, 19 PDs (54%) reported that these factors played no role at all, while 11 PDs (31%) reported them playing a minor role.

### Applicant Feedback

#### Demographics

Forty medical trainees out of 284 (14%) who applied in the 2020 to 2021 CaRMS process and successfully matched into a Canadian surgical residency program responded to the survey: 25 were from Quebec (62.5%), 10 from Ontario (25%), and 1 from Manitoba, Saskatchewan, Alberta, New Brunswick, and Newfoundland and Labrador (2.5% each). The most common specialty applied to was general surgery (n = 17, 43% of all programs applied to by respondents), followed by plastic surgery (n = 6, 15%) and urology (n = 6, 15%). Eighteen respondents (45%) ranked their home school first, and 24 (60% of all participants) matched to their first choice. A detailed breakdown of demographic data is presented in Table 2.

**Table 2.** Demographics of Applicants Participating in the Study.

		Number of students (n)	%
Province	Quebec	25	62.5
	Ontario	10	25
	Saskatchewan	1	2.5
	Manitoba	1	2.5
	Alberta	1	2.5
	New Brunswick	1	2.5
	Newfoundland and Labrador	1	2.5
Surgical Specialties Applied To	General Surgery	17	42.5
	Plastic Surgery	6	15
	Urology	6	15
	Otolaryngology/Head/Neck	5	12.5
	Orthopedic Surgery	4	10
	Opthalmology	3	7.5
	Neurosurgery	3	7.5
	Vascular Surgery	3	7.5
	Cardiac Surgery	2	5
Match to home school	Yes	20	50
	No	20	50
Match to first choice	Yes	24	60
	No	16	40
First choice was home school	Yes	18	55
	No	22	45

Among applicants whose first choices were their home schools (n = 18, 45%), the most commonly reported reasons included geographic preference (n = 14, 74%) and personal life-related matters (family, partner, etc; n = 14, 74%). Elective experience subsequently followed, (n = 13, 68%), as well as “preference to stay in same location” and “institutional reputation” (n = 11, 58% each). For those whose first choices were not their home schools (n = 22, 55%), geographic reasons remained the most common reason for their submitted rankings (n = 14, 64%). This was followed by available clinical opportunities (n = 10, 46%) and perceived work environment (n = 8, 36%). A breakdown of all reported reasons for first-choice ranking by applicants is presented in Supplemental Digital Content 5.

#### Overall applicant confidence in the virtual selection process

Applicants reported an average score of 6.9/10 regarding their perceived confidence in the virtual selection process, with an average score of 7.7/10 regarding their perceived suitability to their first choice-ranked program. A significant association was demonstrated between confidence in suitability with first-choice programs, as well as knowledge of matched programs, with applicants who ranked their home programs first (*P* = .034, and *P* = .005, respectively). When asked about elements of the virtual selection process that helped best-familiarize applicants with programs in the absence of visiting electives, virtual information sessions were most-commonly cited (n = 36, 90%), followed by updated program websites (n = 23, 58%). Only 11 applicants (27.5%) reported a preference for in-person interviews, whereas 29 (72.5%) preferred virtual interviews or were equivocal. Moreover, more than half of applicants (n = 24, 56%) disagreed with the idea that an in-person interview would have allowed them a better chance of matching.

#### Virtual information sessions

All applicants (n = 40, 100%) reported having attended virtual information sessions or virtual events hosted by programs they were interested in. Applicants reported an average score of 7.0/10 regarding the ability of virtual information sessions to increase their interest in programs they may have otherwise not been interested in. Structured presentations from program directors (n = 33, 85%) and informal opportunities to speak with residents (n = 30, 77%) were aspects of the virtual information sessions most appreciated by applicants. When asked about what they would have liked to hear more about during these sessions, 90% of applicants (n = 36) reported an interest in learning more about the work culture and environment, while 34 (85%) were interested in learning about social relationships between residents and staff (Table 3).

**Table 3.** Valued Elements of Virtual Information Sessions for Canadian Surgical Postgraduate Training Program Promotion, Based on Applicant Feedback.

		**n**	**%**
**Format**	Structured presentation from program director	33	83
	Informal opportunities to talk to residents	30	75
	Ability to observe interactions within the team in a virtual context	24	60
	Question and answer period	19	48
	Presentation from research director	8	20
**Content**		**n**	**%**
	Overall work culture and environment	36	90
	Resident-staff interactions	34	85
	Resident-resident relationships	31	78
	Resident work/life balance	27	68
	Clinical opportunities in the program	24	60
	Research/clinical balance	17	43
	Research opportunities in the program	15	38

#### Preinterview socials

When asked about whether preinterview socials helped applicants get to know programs better, n = 34 (86%) responded either “a little bit” or “somewhat.” With regard to suggestions for content and format for these events, applicants overwhelmingly suggested that resident/resident and resident/staff relationships be discussed (n = 38, 95%), followed by discussions about work culture and environment (n = 36, 95%), as seen with virtual information sessions (Supplemental Digital Content 6). With regard to format, casual interactions with residents and staff (n = 33, 83%) were preferred, in addition to icebreaker games and “get-to-know you” activities (n = 23, 58%). Applicants mainly suggested socials be held the day before interviews (n = 29, 76%).

#### Virtual interview

**Figure 1.**
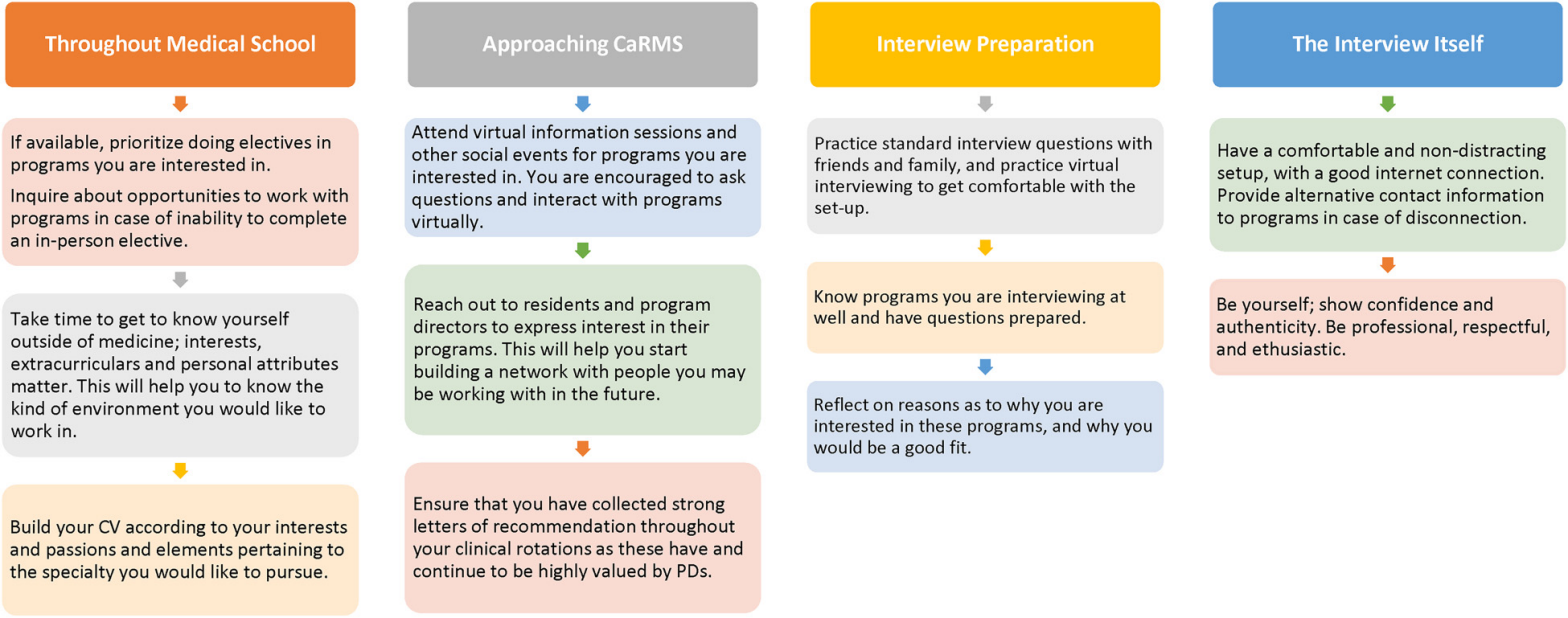
Longitudinal recommendations for medical students applying to Canadian surgical subspecialty programs based on collected feedback from applicants and program directors.

With regard to the virtual interview, 26 respondents (65%) said they appreciated closer interactions with program directors and staff members, and n = 25 (62.5%) reported appreciating a preamble and/or presentation by PDs. With regard to confidence in their ability to properly present their strengths and candidature in the online interview, applicants gave an average answer of 7.45/10, whereas a score of 7.3/10 was given for confidence in ranking programs following virtual interviews. Applicants were asked about aspects of their experiences with the virtual residency selection process that they believe may have contributed to their successful match and were queried for recommendations for future applicants. A summary of key points is synthesized and presented in Figure 1.

## Discussion

The Canadian Resident Matching Service (CaRMS) process has been consistently described as a great source of stress for medical students applying for surgical subspecialties, especially given the growing limitations on available positions.^4^ From its initiation in 1969, until just before the COVID-19 pandemic, Canadian medical graduates applying to a residency program have been traditionally required to perform in-person electives at programs in which they were interested, and to travel across the country for interviews. Despite its institution over these years, this process had become veiled with challenges for both applicants and program directors alike. Previous initiatives have been ascribed on behalf of residency programs to reduce interview burden on Canadian applicants; for example, in 2003, Grober et al described a unified urology fair for information about individual programs and interviews on the same day in the same Canadian city.^5^ The economic impact on applicants, time required for travel, and environmental implication of thousands of applicants traveling for interviews represented just a few reasons that the CaRMS process could have transformed into a virtual format earlier on. However, with the advent of the COVID-19 pandemic, the 2021 CaRMS selection process was forced to become virtual, and a paradigm shift for resident selection in Canada began.^6^

The present study provides both applicants and program directors participating in future iterations of the virtual residency selection process with the perspectives of successful applicants and PDs from the 2021 match, as well as feedback and advice for the improvement of the experience for both applicants and PDs. In addition to the fundamental benefits of curbing COVID-19 spread, the present study demonstrates that there exist several advantages to a virtual format in surgical resident selection. Reduction in stress for applicants and the convenience of being able to interview from the comfort of their homes represent notable advantages. This also serves to increase applicant access to their wellness resources, whether spending quality time with family/friends during the interview period, having access to their traditional facilities for physical activity, or university wellness advisors. With the associated reduction in time required for travel, applicants also reported on the ability to attend more interviews which otherwise may have conflicted by overlapping across different cities. Additionally, the estimated environmental impact of travel during the traditional CaRMS process is nonnegligible, with the virtual switch representing a 100% reduction in greenhouse gas emissions related to this process.^7^ At a time when climate change remains of increasing concern, this itself calls into question whether the travel component for interviews was, at all previously truly warranted.

Among the 40 successful applicants surveyed, a mean response of 7.6/10 and a mode of 9/10 were reported regarding applicant confidence in the knowledge of their matched programs. Although control data from previous years during the traditional match process is not available for the purpose of comparison, these findings demonstrate that overall, despite the virtual format, applicants were still beginning their postgraduate training with significant confidence and knowledge of their programs. Applicant confidence in this context was demonstrated to be statistically correlated with instances of matches to home schools, which may further reinforce the importance of elective opportunities and in-person program exposure, regardless of the interview format, as highlighted by feedback from program directors surveyed in this study. These findings are in line with additional studies from the United States, including on nonsurgical training programs. When asked about preferences regarding future recruitment strategies to pediatric neurology programs, American program directors reported a preference for combined experiences that include in-person social activities or program visits, with continuing online interviews.^8^ Another study on American surgical oncology programs found that most PDs (83%) and candidates (79%) were comfortable making their rank lists following virtual interviews, in line with the remarkably high PD and applicant confidence in ranking and match outcomes following virtual interviews in the present study.^9^ In contrast, a similar study surveying American plastic surgery program directors found that most PDs (76.3%) would not prefer to have virtual interviews in future years, whereas in the present study, findings were rather split regarding PD and student preference.^10^ Ultimately, this accentuates the importance of ongoing discussion regarding the future of residency interviewing and selection in order to leverage the benefits of virtual interviewing and selection, all while minimizing its limitations.

Despite its notable advantages, the virtual residency selection process is not without its limitations. Technological difficulties arising from poor internet connection, accidental disconnection, breakout room confusion, and overall virtual applicant and interviewer coordination were stated as marked disadvantages. Indeed, computer proficiency, literacy, and technological difficulties were cited as notable challenges in recent studies, along with a compromised element of social interaction and loss of opportunity for applicants to observe program environments.^11,12^ Although these limitations may be unique to the virtual applicant process of 2021; reinstitution of in-person visiting electives as part of the virtual residency selection process may circumvent most of these challenges. Although this study did not query the specific impact of the lack of visiting electives on satisfaction with match outcomes among PDs and applicants, the significance of clinical electives for applicant selection was noted by several PDs. Many PDs felt that regardless of selection process format, virtual or in-person, nothing could replace the value of in-person electives; and while 90% felt either “very” or “somewhat” confident in the fit of their selected residents, it remains unclear to what extent the absence of electives impacts this perceived confidence and overall PD satisfaction. In contrast, given that all applicant respondents were those who had matched, naturally, most felt that the absence of electives and virtual format didn't have any impact on their chances of matching to their top programs. The impact of absent clinical electives on perceived satisfaction in surgical candidate selection among PDs remains an interesting consideration for our group, which we hope to address in future studies. Nonetheless, taken together with the ability to increase the flexibility for scheduling, reduced cost for applicants and programs, increased participation of multiple faculty members, and reduced travel for applicants, the advantages of virtual interviewing and applicant recruitment may significantly outweighing limitations of the virtual process presented herein.^11,13^

### Recommendations for applicants

Strong letters and interviews remain the most significant factors in applicant selection, consistent with practices prior to virtual resident selection.^14^ Although attendance to virtual information sessions was not reported by PDs to increase likelihood of being offered an interview, many applicants in the 2021 CaRMS cycle found that attendance helped their ability to get to know programs better and make informed decisions on program suitability. Applicants are encouraged by some PDs to reach out to programs in which they are interested, in order to learn more about them, get involved in research, and perform visiting electives, if and when possible. During the interviews themselves, applicants are encouraged to interact with the entire interview panel and treat virtual interviews as though they were in-person. Although interview setup, lighting, and background choice were not significant factors in applicant selection from the perspective of PDs, choice of a quiet, nondistracting, and professional environment, as well as professional attire, can help better-simulate the traditional interview experience (Figure 1).

### Recommendations for program directors

**Figure 2.**
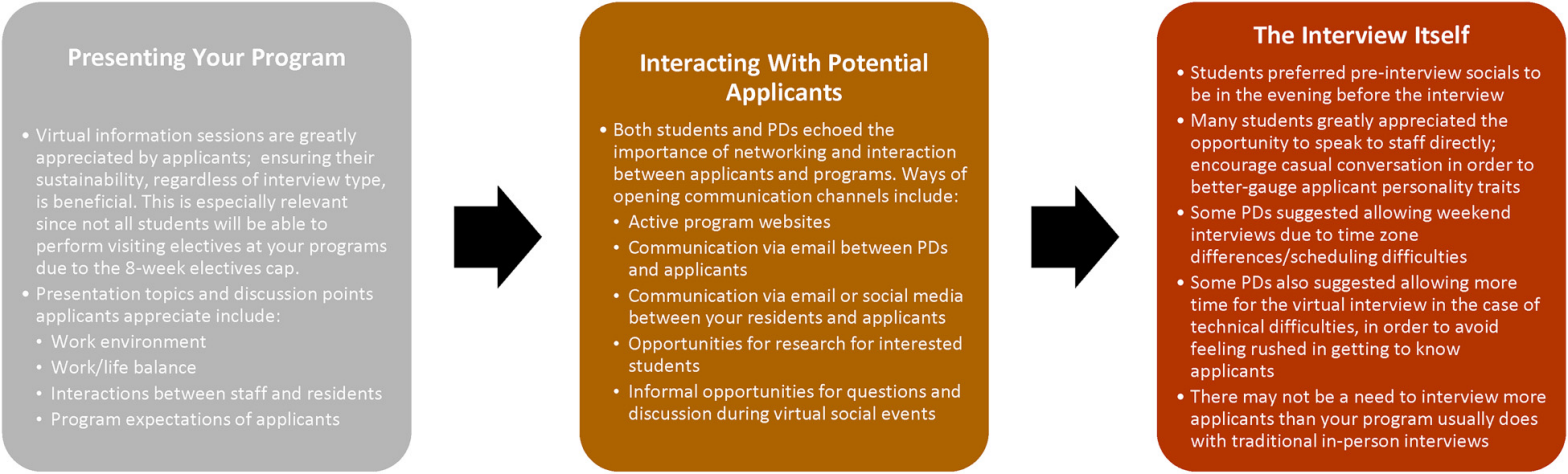
Recommendations for Canadian surgical program directors navigating the Canadian surgical subspecialty resident selection process, based on collected feedback from applicants and program directors.

Virtual information sessions remain highly valued by applicants as useful tools to get to know programs they are unable to visit; ensuring their sustainability, regardless of interview format in future years, can help promote your program. Applicants are interested in learning about the work environment, resident work–life balance, and resident–staff relationships, in addition to academic and clinical highlights of your program. Given the Association of Faculties of Medicine of Canada (AFMC) elective diversification policy that limits electives within one particular specialty to a maximum of 8 weeks, applicants may no longer count on the ability to perform visiting electives at programs in which they are interested, regardless of waning pandemic restrictions.^15,16^ Programs may thus need to reconsider policies in which in-person electives represent a major requirement for offering interviews to applicants. Although programs may consider the option of offering an in-person interview in order to better-interact with these applicants, the potential selection bias that may arise toward applicants afforded in-person interviews cannot be ignored. Opportunities for direct faculty and resident interactions with applicants remain highly valued, whether during the virtual information sessions, social events, or interviews themselves (Figure 2).

### Limitations

This study is not without its limitations. The cross-sectional nature of this study and lack of follow-up, to better understand the longitudinal impact of the 2021 virtual selection process, cannot be ignored. Additionally, a limited sample size of participants was analyzed, with data captured from 35/128 (27%) of Canadian surgical program directors,^17^ and 40/284 (14%) of successful surgical applicants.^18^ Overrepresentation of Quebec applicants in the survey process, given affiliation of study organizers to Quebec home programs, may represent an added limitation. Given that the study captured data from only successful applicants, perceptions of the 2021 CaRMS process may be biased in a positive fashion. Lack of control data from earlier cohorts who participated in the traditional CaRMS process precludes strong comparative conclusions. Nonetheless, the conclusions and advice presented herein remain useful as a guide for both applicants and PDs on ways to better-navigate upcoming virtual surgical residency selection processes, during the time of COVID-19, and beyond.

## Conclusions

Trainees are entering residency with confidence following virtual interviews, and PDs feel confident in their selections. Although no clear consensus exists regarding preference for a virtual or in-person interview, several advantages for virtual selection exist. The influence of an in-person elective may be difficult to replace, regardless of interview type. The importance of applicant engagement with programs prior to interviews is highlighted and discussed, with guidelines for best practices presented. With surgical residency interviews remaining online despite waning COVID-19 restrictions, PDs and applicants must continuously adapt recruitment and engagement methods in order to optimize their match outcomes.

## Supplemental Material

sj-docx-1-psg-10.1177_22925503221108468 - Supplemental material for Successful Applicant and Program Director Perspectives on the Virtual Residency Selection Process for Canadian Surgical Subspecialties Supplemental material, sj-docx-1-psg-10.1177_22925503221108468 for Successful Applicant and Program Director Perspectives on the Virtual Residency Selection Process for Canadian Surgical Subspecialties by Jad Abi-Rafeh, Victoria Sebag and Hassan ElHawary, Dino Zammit, Mirko S. Gilardino in Plastic Surgery

sj-pdf-2-psg-10.1177_22925503221108468 - Supplemental material for Successful Applicant and Program Director Perspectives on the Virtual Residency Selection Process for Canadian Surgical Subspecialties Supplemental material, sj-pdf-2-psg-10.1177_22925503221108468 for Successful Applicant and Program Director Perspectives on the Virtual Residency Selection Process for Canadian Surgical Subspecialties by Jad Abi-Rafeh, Victoria Sebag and Hassan ElHawary, Dino Zammit, Mirko S. Gilardino in Plastic Surgery

sj-pdf-3-psg-10.1177_22925503221108468 - Supplemental material for Successful Applicant and Program Director Perspectives on the Virtual Residency Selection Process for Canadian Surgical Subspecialties Supplemental material, sj-pdf-3-psg-10.1177_22925503221108468 for Successful Applicant and Program Director Perspectives on the Virtual Residency Selection Process for Canadian Surgical Subspecialties by Jad Abi-Rafeh, Victoria Sebag and Hassan ElHawary, Dino Zammit, Mirko S. Gilardino in Plastic Surgery

sj-pdf-4-psg-10.1177_22925503221108468 - Supplemental material for Successful Applicant and Program Director Perspectives on the Virtual Residency Selection Process for Canadian Surgical Subspecialties Supplemental material, sj-pdf-4-psg-10.1177_22925503221108468 for Successful Applicant and Program Director Perspectives on the Virtual Residency Selection Process for Canadian Surgical Subspecialties by Jad Abi-Rafeh, Victoria Sebag and Hassan ElHawary, Dino Zammit, Mirko S. Gilardino in Plastic Surgery

sj-pdf-5-psg-10.1177_22925503221108468 - Supplemental material for Successful Applicant and Program Director Perspectives on the Virtual Residency Selection Process for Canadian Surgical Subspecialties Supplemental material, sj-pdf-5-psg-10.1177_22925503221108468 for Successful Applicant and Program Director Perspectives on the Virtual Residency Selection Process for Canadian Surgical Subspecialties by Jad Abi-Rafeh, Victoria Sebag and Hassan ElHawary, Dino Zammit, Mirko S. Gilardino in Plastic Surgery

sj-pdf-6-psg-10.1177_22925503221108468 - Supplemental material for Successful Applicant and Program Director Perspectives on the Virtual Residency Selection Process for Canadian Surgical Subspecialties Supplemental material, sj-pdf-6-psg-10.1177_22925503221108468 for Successful Applicant and Program Director Perspectives on the Virtual Residency Selection Process for Canadian Surgical Subspecialties by Jad Abi-Rafeh, Victoria Sebag and Hassan ElHawary, Dino Zammit, Mirko S. Gilardino in Plastic Surgery

sj-pdf-7-psg-10.1177_22925503221108468 - Supplemental material for Successful Applicant and Program Director Perspectives on the Virtual Residency Selection Process for Canadian Surgical Subspecialties Supplemental material, sj-pdf-7-psg-10.1177_22925503221108468 for Successful Applicant and Program Director Perspectives on the Virtual Residency Selection Process for Canadian Surgical Subspecialties by Jad Abi-Rafeh, Victoria Sebag and Hassan ElHawary, Dino Zammit, Mirko S. Gilardino in Plastic Surgery
