# Supplementary material for: Successful Applicant and Program Director Perspectives on the Virtual Residency Selection Process for Canadian Surgical Subspecialties
Source: Plast Surg (Oakv). 2022 Jul 5;32(2):339–46. doi: 10.1177/22925503221108468 (PMC11046273; doi:10.1177/22925503221108468)
Supplement: sj-docx-1-psg-10.1177_22925503221108468 - Supplemental material for Successful Applicant and Program Director Perspectives on the Virtual Residency Selection Process for Canadian Surgical Subspecialties [file sj-docx-1-psg-10.1177_22925503221108468.docx]

**Supplementary Content Legend:**

**Supplemental Digital Content 1**

*Supplementary Methods*

**Supplemental Digital Content 2**

*Survey administered to applicants participating in the 2021 virtual CaRMS selection process for surgical subspecialties.*

**Supplemental Digital Content 3**

*Survey administered to program directors participating in the 2021 virtual CaRMS selection process for surgical subspecialties.*

**Supplemental Digital Content 4**

*Supplementary Table 1: Number of surgical program directors who responded to the survey as a percentage of total number of surgical program directors, per province*

**Supplemental Digital Content 5**

*Supplementary Table 2: Reasons for trainee ranking of first-choice programs*

**Supplemental Digital Content 6**

*Supplementary Table 3: Preferred discussion content during pre-interview social events based on feedback from applicants*
