# Supplementary material for: Successful Applicant and Program Director Perspectives on the Virtual Residency Selection Process for Canadian Surgical Subspecialties
Source: Plast Surg (Oakv). 2022 Jul 5;32(2):339–46. doi: 10.1177/22925503221108468 (PMC11046273; doi:10.1177/22925503221108468)
Supplement: sj-pdf-2-psg-10.1177_22925503221108468 - Supplemental material for Successful Applicant and Program Director Perspectives on the Virtual Residency Selection Process for Canadian Surgical Subspecialties [file sj-pdf-2-psg-10.1177_22925503221108468.pdf]

Supplemental Digital Content 1 - Supplementary Methods

The questionnaire was designed using Google Forms; 1-10 Likert scales as well as "select all that apply" options were used for responses. The latter option was used for qualitative questions, whereas the Likert scale was used mainly to quantify degrees of confidence and feedback regarding different engagement methods. Questions for applicants queried their experience with virtual methods of engagement across different programs, including virtual information sessions, pre-interview social events, and the CaRMS interview itself. For virtual information sessions and pre-interview socials, applicants were asked about which event format and discussion topics were most appreciated. With regards to interviews, applicants were queried about aspects in the interview that helped them get to know programs better, and feel more comfortable in their subsequent ranking. The full applicant survey is presented in **Supplemental Digital Content 2**.

The questionnaire for program directors inquired about aspects that made candidates more attractive to residency selection committees in the absence of having done a visiting elective. Program directors' perspective on advantages and disadvantages of the online process, preferred recruitment methods, and recommendations for improvement of the virtual selection process were also solicited. The full program director survey is presented in **Supplemental Digital Content 3**.

<u>Analysis</u>

Responses that were answered with Likert-scales were analyzed quantitatively and presented as percentages, proportions, and means, where appropriate. A Pearson correlation analysis was conducted to identify significant associations between responses and different cohorts, where appropriate. Qualitative responses or comments were coded using Microsoft Excel and presented as trends within participant answers.
