# Supplementary material for: Successful Applicant and Program Director Perspectives on the Virtual Residency Selection Process for Canadian Surgical Subspecialties
Source: Plast Surg (Oakv). 2022 Jul 5;32(2):339–46. doi: 10.1177/22925503221108468 (PMC11046273; doi:10.1177/22925503221108468)
Supplement: sj-pdf-3-psg-10.1177_22925503221108468 - Supplemental material for Successful Applicant and Program Director Perspectives on the Virtual Residency Selection Process for Canadian Surgical Subspecialties [file sj-pdf-3-psg-10.1177_22925503221108468.pdf]

Supplemental Digital Content 2

Survey administered to applicants participating in the 2021 virtual CaRMS selection process for surgical subspecialties.

2021 Residency Match - Online Process

To medical trainees who applied in the CARMs process this year: Thank you for taking the time to answer our survey on the 2021 CARMs process, which took place virtually this year. We hope to use your answers to assess whether the process was preferred this way and to make recommendations for future years.

* Required

Consent Form

Project Title: Student and Program Director Perspective on Virtual Interviewing in the CaRMS Match Process.

Principal Investigator: Mirko S. Gilardino MD, MSc, FRCSC, FACS

Sponsors/Financial compensation: None

Purpose of the study: The primary goal of this study is to assess overall confidence with the virtual Canadian Resident Matching Service (CaRMS) process for surgical specialties, including interviews, pre-interview socials, as well as virtual information sessions due to the COVID-19 pandemic. Secondary goals of this study include seeking to provide recommendations to programs as to how to enhance the virtual process for applying students, and to provide students with advice as to how to navigate the virtual process. By analyzing these factors, future generations of medical students can better-prepare themselves for their CaRMS applications, and programs can provide insight as to how to improve the overall process.

Study procedure: You will be presented with a 10-20 minute questionnaire (16 questions for program directors, 31 questions for students) inquiring about your experiences with the 2021 CARMs process. The questionnaire can be answered at your convenience on your personal computer.

Benefits associated to your participation: Although participation in this study does not benefit you directly, it will help improve the virtual CARMs process, in turn benefitting both residency programs and applying trainees in future years.

Disadvantages and risks that may arise from your participation: There is no foreseeable risks for participation in this study. If at any points you felt uncomfortable completing the questionnaire, please feel free to close the web browser. Right of withdrawal without prejudice: It is understood that your participation in this research project is completely voluntary and that you remain free, at any time, to end your participation without having to explain your decision or to undergo harm of any kind. No identifying information will be asked.

Confidentiality, Sharing, Monitoring and Publications: Following completion of the questionnaires, your answers will be stored on a secure cloud-based database without any association with your personal information. Only information necessary for the proper conduct of the research project will be collected. All information collected during the research project will be kept strictly confidential within the limits of the law. In order to preserve the confidentiality of this information, you will not be identified in this anonymous questionnaire. The principal investigators of the study will use the data for research purposes in order to meet the scientific objectives of the research project described in this consent form. Research project data may be published in scientific journals or shared with others in scientific discussions. No publication or scientific communication will contain any information that may identify you.

Right of withdrawal without prejudice: It is understood that your participation in this research project is completely voluntary and that you remain free, at any time, to end your participation without having to explain your decision or to undergo harm of any kind. Please note that since the survey contains no identifying questions, once the survey is completed and submitted it is impossible to discard your answers.

1. I have read and completely understand the information included above. I voluntarily agree to participate in this study and to proceed to answering the questionnaire.

- Yes
- No

If you refuse to provide consent, you can close your browser without having to answer the questionnaire below. If you are ready to complete the survey, please press 'next'.

Survey

2. How old are you?*

3. Which gender do you identify with?*
- Female
- Male
- Non-binary
- Prefer not to say
- Other:

4. Which province is your medical school located in?*
- British Columbia
- Alberta
- Saskatchewan
- Manitoba
- Ontario
- Quebec
- Newfoundland and Labrador
- New Brunswick
- Nova Scotia
- PEI

5. Which surgical specialty(ies) did you apply to this year?*
- Cardiac Surgery
- General Surgery
- Neurosurgery
- Opthalmology
- Orthopedic Surgery
- Plastic Surgery
- Urology
- Vascular Surgery
- Otolaryngology / Head and Neck Surgery / ENT
- Obstetrics & Gynecology

6. Did you match to a residency program?*
- Yes
- No

7. Did you match to your home school?*
- Yes
- No

8. Did you match to your first choice?*
- Yes
- No

9. Was your home school your first choice?*
- Yes
- No

10. If yes to previous question, why? (select all that apply and/or specify with "other")
- Prefer to stay in same location
- Institutional reputation
- Elective experience
- Research opportunities
- Clinical opportunities
- Established mentorship
- Love of city in which program is located
- Personal life-related reasons (family, partner, etc)
- Useful virtual information session (if attended)
- Important information diffused using alternative methods (social media, outreach, virtual events)
- Work environment
- Other:

11. If your first choice was not your home school, why? (select all that apply and/or specify with "other")
- Institutional reputation
- Elective experience
- Research opportunities
- Clinical opportunities
- Established mentorship
- Location/Geography
- Useful virtual information session (if attended)
- Important information diffused using alternative methods (social media, outreach, virtual events)
- Work environment
- Other:

12. Did you attend any virtual information information sessions or events for programs you were interested in applying to?*
- Yes
- No

13. Did virtual information sessions increase your confidence or interest in programs you may have not initially been interested in?

Not at all

- 1
- 2
- 3
- 4
- 5
- 6
- 7
- 8
- 9
- 10
- Completely

14. Which aspects of the virtual information sessions helped you to feel comfortable in your knowledge of the program? (select all that apply and/or specify with "other")

- Structured presentation from program director
- Presentation from research director
- Informal opportunities to talk to residents (ice breaker games, casual discussion)
- Ability to observe interactions within the team in virtual context
- Q&A periods
- Other:

15. What did you/would you appreciate these information sessions to discuss to increase confidence applying to a program? (select all that apply and/or specify with "other")*

- Resident-resident relationships
- Resident-Staff interactions
- Research opportunities in program
- Clinical opportunities in program
- Research/Clinical balance
- Overall work culture and environment
- Work/life balance
- Other:

16. How well do you feel that you got to know the programs you interviewed at (and their residents) through the pre-interview socials?*

- Not at all
- A little bit
- Somewhat
- Very much

17. Pre-Interview Social Timing: What timing of the pre-interview social did you find most convenient for getting to know the program and its team, in the context of the busy interview schedule? (select all that apply and/or specify with "other")*

- Right before the interview
- Day before the interview
- Morning
- Evening
- Afternoon
- Other:

18. Pre-Interview Social Content: What content of the pre-interview social did you find most useful for getting to know the program and its team? (select all that apply and/or specify with "other")*

- Resident/resident staff/staff resident/staff relationships
- Research opportunities
- Clinical opportunities
- Research/Clinical Balance
- Program expectations
- Work/life balance
- Work environment and culture
- Information about program location (sightseeing, weather, etc)
- Other:

19. Pre-Interview Social Format: What format of the pre-interview social did you find most useful for getting to know the program and its team? (select all that apply and/or specify with "other")*

- Format: Casual interactions with residents/potential future coworkers
- Format: Formal presentations
- Format: Q&A Period
- Format: Icebreaker/get-to-know you activities
- Other:

20. What aspects of the virtual interview itself helped (or would help) you get to know the program better? (select all that apply and/or specify with "other")*

- Closer interaction with Program Director/staff members
- Opportunity for 1on1 discussion
- Opportunity to ask questions directly

- Presentation/preamble from program director or staff members
- Other:

21. Through the online interview, do you feel you were able to properly present yourself and your strengths as a candidate?*

Not at all

- 1
- 2
- 3
- 4
- 5
- 6
- 7
- 8
- 9
- 10

Completely

22. How comfortable were you ranking different programs after interviewing with them? *

Not at all

- 1
- 2
- 3
- 4
- 5
- 6
- 7
- 8
- 9
- 10

Completely

23. How confident were you that your first choice was the best fit for you?*

Not at all

- 1
- 2
- 3
- 4
- 5
- 6
- 7
- 8
- 9
- 10

Completely

24. Preference for virtual interview?*

- Yes
- No
- Unsure/No preference

25. Confidence in the virtual interview selection process *

Not at all

- 1
- 2
- 3
- 4
- 5
- 6
- 7
- 8
- 9
- 10

Completely confident

26. Do you agree with: “an in person interview would have allowed me a better chance of matching / matching to a higher choice on my rank list”*

- Yes
- No
- Unsure

27. If you matched to a surgical program, how confident are you starting your program (knowledge of the program, what to expect, work environment)?

Not at all

- 1
- 2
- 3
- 4
- 5
- 6
- 7
- 8
- 9
- 10

Completely

28. What helped you in getting to know programs best in the absence of having done a clinical elective with them? (select all that apply and/or specify with "other")*

- Virtual information session
- Preinterview social event

- Social media presence of programs/residents
- Updated program website
- The interview itself
- Other:

29. If you matched: what do you feel contributed to your successful match during the virtual interview process? [long answer]

30. If you matched: what are your recommendations for future applicants navigating the virtual interview process? [long answer]

31. If you did not match: what aspects of the virtual interview process do you feel contributed to you going unmatched? [long answer]

32. If you did not match: what would you have done differently in hindsight? What would you recommend to future applicants? [long answer]
