# Supplementary material for: Successful Applicant and Program Director Perspectives on the Virtual Residency Selection Process for Canadian Surgical Subspecialties
Source: Plast Surg (Oakv). 2022 Jul 5;32(2):339–46. doi: 10.1177/22925503221108468 (PMC11046273; doi:10.1177/22925503221108468)
Supplement: sj-pdf-4-psg-10.1177_22925503221108468 - Supplemental material for Successful Applicant and Program Director Perspectives on the Virtual Residency Selection Process for Canadian Surgical Subspecialties [file sj-pdf-4-psg-10.1177_22925503221108468.pdf]

Supplemental Digital Content 3

Survey administered to to program directors participating in the 2021 virtual CaRMS selection process for surgical subspecialties.

2021 Residency Match - Online Process

Canadian Surgical Program Directors: Thank you for taking the time to answer our survey on the 2021 CARMs process, which took place virtually this year. We hope to use your answers to assess whether the process was preferred this way and to make recommendations for future years.

Consent

Project Title: Student and Program Director Perspective on Virtual Interviewing in the CaRMS Match Process.

Principal Investigator: Mirko S. Gilardino MD, MSc, FRCSC, FACS

Sponsors/Financial compensation: None

Purpose of the study: The primary goal of this study is to assess overall confidence with the virtual Canadian Resident Matching Service (CaRMS) process for surgical specialties, including interviews, pre-interview socials, as well as virtual information sessions due to the COVID-19 pandemic. Secondary goals of this study include seeking to provide recommendations to programs as to how to enhance the virtual process for applying students, and to provide students with advice as to how to navigate the virtual process. By analyzing these factors, future generations of medical students can better-prepare themselves for their CaRMS applications, and programs can provide insight as to how to improve the overall process.

Study procedure: You will be presented with a 10-20 minute questionnaire (16 questions for program directors, 31 questions for students) inquiring about your experiences with the 2021 CARMs process. The questionnaire can be answered at your convenience on your personal computer.

Benefits associated to your participation: Although participation in this study does not benefit you directly, it will help improve the virtual CARMs process, in turn benefitting both residency programs and applying trainees in future years.

Disadvantages and risks that may arise from your participation: There is no foreseeable risks for participation in this study. If at any points you felt uncomfortable completing the questionnaire, please feel free to close the web browser. Right of withdrawal without prejudice: It is understood that your participation in this research project is completely voluntary and that you remain free, at any time, to end your participation without having to explain your decision or to undergo harm of any kind. No identifying information will be asked.

Confidentiality, Sharing, Monitoring and Publications: Following completion of the questionnaires, your answers will be stored on a secure cloud-based database without any association with your personal information. Only information necessary for the proper conduct of the research project will be collected. All information collected during the research project will be kept strictly confidential within the limits of the law. In order to preserve the confidentiality of this information, you will not be identified in this anonymous questionnaire. The principal investigators of the study will use the data for research purposes in order to meet the scientific objectives of the research project described in this consent form. Research project data may be published in scientific journals or shared with others in scientific discussions. No publication or scientific communication will contain any information that may identify you.

Right of withdrawal without prejudice: It is understood that your participation in this research project is completely voluntary and that you remain free, at any time, to end your participation without having to explain your decision or to undergo harm of any kind. Please note that since the survey contains no identifying questions, once the survey is completed and submitted it is impossible to discard your answers.

1. I have read and completely understand the information included above. I voluntarily agree to participate in this study and to proceed to answering the questionnaire.
Mark only one oval.
   - Yes
   - No

If you refuse to provide consent, you can close your browser without having to answer the questionnaire below. If you are ready to complete the survey, please press 'next'.

Survey

2. Please Select Your Program
- Cardiac Surgery
- General Surgery
- Neurosurgery
- Opthalmology
- Orthopedic Surgery
- Otolaryngology - Head and Neck Surgery
- Plastic Surgery
- Urology
- Vascular Surgery

3. Please select your program's province
- British Columbia
- Alberta
- Saskatchewan
- Manitoba
- Ontario
- Quebec
- Newfoundland and Labrador
- New Brunswick
- Nova Scotia
- PEI

4. How many residents were you able to select for your program through the CaRMS matching process? [short answer]

5. How well do you feel you were able to gauge applicants and their personalities through the virtual interview process?
- Not at all
- A little bit
- Somewhat
- Very much

6. What methods did your program use to recruit and interact with potential applicants to your program? (select all that apply and/or specify with "other")
- Virtual Information Sessions
- Virtual social events with residents
- Active social media pages
- Active program website
- Open communication with yourself and applicants via email
- Other:

7. How well do you feel you were able to represent your program using virtual methods (social media, information sessions, etc)?
- Not at all
- A little bit
- Somewhat
- Very much

8. How confident are you in the fit of residents matched to your program, relative to previous years in which in-person interviews were offered?
- Not at all
- A little bit
- Somewhat
- Very much

9. For you, what were the benefits of online interviews? (select all that apply and/or specify with "other")
- Cost reduction
- Efficiency
- Greater number of interviews conducted
- Less resources and planning needed
- Other:

10. For you, what were the disadvantages of online interviews? (select all that apply and/or specify with "other")
- Less ability to socialize with applicants and gauge their personalities and interpersonal traits
- Higher volume interviewed unnecessarily
- No opportunity for applicant to tour faculty
- No opportunity for in-person socials
- Other:

11. For you, did the benefits of online interviews outweigh the disadvantages?
- Yes
- No

12. What factors made applicants from outside your home school more appealing given the inability to complete an elective with your program? (select all that apply and/or specify with "other")
- Strong letters
- Research experience
- Strong interview, preparedness
- Extracurricular activities (non-medicine)
- Leadership positions
- Other:

13. Did student attendance to virtual information sessions or events given by your program increase their likelihood of being invited to interview for your program?

- Not at all
- A little bit
- Somewhat
- Very much

14. Did external factors such as lighting, background and clothing choice for the virtual interview process play any role in the selection process, and if so, to what extent?

- Not at all
- A little bit
- Somewhat
- Very much

15. Would you like for interviews to be conducted virtually for future cohorts?

- Yes
- No

16. What suggestions do you have for improving the virtual interview process for future years, and making it more sustainable? [long answer]

17. What are your recommendations for online interviewing for future applicants to your program? [long answer]
