# Supplementary material for: Successful Applicant and Program Director Perspectives on the Virtual Residency Selection Process for Canadian Surgical Subspecialties
Source: Plast Surg (Oakv). 2022 Jul 5;32(2):339–46. doi: 10.1177/22925503221108468 (PMC11046273; doi:10.1177/22925503221108468)
Supplement: sj-pdf-5-psg-10.1177_22925503221108468 - Supplemental material for Successful Applicant and Program Director Perspectives on the Virtual Residency Selection Process for Canadian Surgical Subspecialties [file sj-pdf-5-psg-10.1177_22925503221108468.pdf]

Supplemental Digital Content 4

Supplementary Table 1: Number of surgical program directors who responded to the survey as a percentage of total number of surgical program directors, per province

	Total PDs for surgical programs (n)	PDs responded to survey (n)	PD response by province (%)
Quebec	29	13	45
Ontario	46	7	15
Alberta	15	5	33
Saskatchewan	6	4	66
Nova Scotia	8	3	38
British Columbia	10	2	20
Manitoba	10	1	10
