# Supplementary material for: Successful Applicant and Program Director Perspectives on the Virtual Residency Selection Process for Canadian Surgical Subspecialties
Source: Plast Surg (Oakv). 2022 Jul 5;32(2):339–46. doi: 10.1177/22925503221108468 (PMC11046273; doi:10.1177/22925503221108468)
Supplement: sj-pdf-6-psg-10.1177_22925503221108468 - Supplemental material for Successful Applicant and Program Director Perspectives on the Virtual Residency Selection Process for Canadian Surgical Subspecialties [file sj-pdf-6-psg-10.1177_22925503221108468.pdf]

Supplemental Digital Content 5

Supplementary Table 2: Reasons for trainee ranking of first-choice programs

		n	%
**Home School as First Choice**			
	Love of city in which program is located	14	73.68
	Personal life-related reasons (family, partner, etc)	14	73.68
	Elective experience	13	68.42
	Institutional reputation	11	57.89
	Prefer to stay in same location	11	57.89
	Clinical opportunities	10	52.63
	Established Mentorship	10	52.63
	Work environment	7	36.84
	Research opportunities	5	26.32
	Useful virtual information session	2	10.53
	Important information diffused using alternative methods	1	5.26
**Other School than Home School as First choice**		**n**	**%**
	Location/geography	14	63.64
	Clinical opportunities	10	45.45
	Work environment	8	36.36
	Institutional reputation	6	27.27
	Research opportunities	5	22.73
	Useful virtual information session	5	22.73
	Research opportunities	5	22.73
	Personal life-related reasons (family, partner, etc)	3	13.64
	Important information diffused using alternative methods	3	13.64
	Only interview received	2	9.09
	Established mentorship	1	4.55
