# Supplementary material for: Successful Applicant and Program Director Perspectives on the Virtual Residency Selection Process for Canadian Surgical Subspecialties
Source: Plast Surg (Oakv). 2022 Jul 5;32(2):339–46. doi: 10.1177/22925503221108468 (PMC11046273; doi:10.1177/22925503221108468)
Supplement: sj-pdf-7-psg-10.1177_22925503221108468 - Supplemental material for Successful Applicant and Program Director Perspectives on the Virtual Residency Selection Process for Canadian Surgical Subspecialties [file sj-pdf-7-psg-10.1177_22925503221108468.pdf]

Supplemental Digital Content 6

Supplementary Table 3: Preferred discussion content during pre-interview social events based on feedback from applicants

	n	%
Resident/resident, resident/staff relationships	38	95
Work environment and culture	36	90
Work/life balance	29	72.5
Program expectations	25	62.5
Clinical opportunities	16	40
Information about program location (sightseeing, weather)	16	40
Research opportunities	11	27.5
Research/clinical balance	7	17.5
